# Contrasting the Effects of Maternal and Behavioral Characteristics on Fawn Birth Mass in White-Tailed Deer

**DOI:** 10.1371/journal.pone.0136034

**Published:** 2015-08-19

**Authors:** Eric S. Michel, Stephen Demarais, Bronson K. Strickland, Jerrold L. Belant

**Affiliations:** 1 Department of Wildlife, Fisheries and Aquaculture, Forest and Wildlife Research Center, Deer Ecology and Management Laboratory, Mississippi State University, Mississippi State, Mississippi, United States of America; 2 Carnivore Ecology Laboratory, Forest and Wildlife Research Center, Mississippi State University, Mississippi State, Mississippi, United States of America; University of Jyväskylä, FINLAND

## Abstract

Maternal care influences offspring quality and can improve a mother’s inclusive fitness. However, improved fitness may only occur when offspring quality (i.e., offspring birth mass) persists throughout life and enhances survival and/or reproductive success. Although maternal body mass, age, and social rank have been shown to influence offspring birth mass, the inter-dependence among these variables makes identifying causation problematic. We established that fawn birth mass was related to adult body mass for captive male and female white-tailed deer (*Odocoileus virginianus*), thus maternal care should improve offspring fitness. We then used path analysis to identify which maternal characteristic(s) most influenced fawn birth mass of captive female white-tailed deer. Maternal age, body mass and social rank had varying effects on fawn birth mass. Maternal body mass displayed the strongest direct effect on fawn birth mass, followed by maternal age and social rank. Maternal body mass had a greater effect on social rank than age. The direct path between social rank and fawn birth mass may indicate dominance as an underlying mechanism. Our results suggest that heavier mothers could use dominance to improve access to resources, resulting in increased fitness through production of heavier offspring.

## Introduction

Maternal phenotype can have profound implications on life history traits. Maternal body mass can influence individual longevity [1] and maternal age and body mass can influence offspring characteristics such as parturition date, growth rate and body size at maturity [2]. Similarly, maternal body mass can positively influence the probability of reproduction [3], litter size [4, 5] and ability to provide increased pre- and postnatal care [4, 6]. The positive influence of maternal phenotype on prenatal care can also influence offspring quality as older and heavier mothers generally produce heavier offspring with increased growth rates compared to younger and lighter mothers [7–9]. Generally, heavier mothers obtain fitness advantages over the long term when compared to lighter mothers [1].

Maternal behavior may complement, or compensate for, the effect of maternal phenotype on life history traits. For example, boldness [10–13] and territoriality [13] can positively influence reproduction, weaning success, survival and growth rate. Dominance also increases food acquisition and time spent feeding within [14] and among selected forage species [15]. Such behaviors are advantageous during times of food limitation [16] and abundance [12, 14]. Thus, maternal behavior can also positively influence offspring quality [17].

Producing high-quality offspring is largely beneficial if offspring retain quality throughout life. In large mammals, offspring quality is positively related to their adult phenotype as heavy offspring had greater body mass and larger secondary sexual characteristics (e.g., horn length) when compared to light offspring [18, 19]. Secondary sexual characteristics can positively influence reproduction [20–22]; therefore, in stable environments the ability of a mother to produce high-quality offspring is beneficial as they may display increased fitness when compared to low-quality offspring [23]. Simultaneous evaluation of maternal phenotypic characteristics and individual behaviors may allow identification of morphometric features associated with, and mechanisms used by mothers to improve offspring quality.

Our first goal was to establish if heavy birth mass persisted to maturity for captive male and female white-tailed deer (*Odocoileus virginianus*). Body mass early in life has been shown to be related to body mass later in life [18, 19]; therefore, we predicted that fawn birth mass would be positively related to mature mass (≥ 3 years of age). Next, we simultaneously assessed how maternal phenotypic and behavioral characteristics affected offspring birth mass for captive white-tailed deer. Maternal body mass and age have been shown to influence fawn birth mass as older and larger mothers have more resources to allocate to reproduction compared to younger and smaller mothers [4, 5]. Thus, we predicted that maternal body mass and age would have positive effects on fawn birth mass. Additionally, we assumed that increased social rank position confers a benefit as increased rank position leads to increased access to food when limited, thus improving body condition and potentially reproductive success [24–26]. However, these benefits may be reduced when food is abundant [27, 28], so we predicted that social rank would minimally affect fawn birth mass due to our *ad libitum* feeding regimen.

## Materials and Methods

### Ethics statement

The Mississippi State University Institutional Animal Care and Use Committee approved all capture, handling, and marking techniques under protocols 04–068, 07–036, 10–033 and 13–034. Mississippi Department of Wildlife, Fisheries and Parks and private landowners approved sampling locations and procedures for this research. Sampling procedures did not involve endangered species.

### Study area

We conducted this research at the Johnnie R. “Rusty” Dawkins Memorial Deer Unit at Mississippi State University (MSU Deer Unit), a 4.9-ha facility consisting of 5 pens (0.4–0.8 ha) located in Oktibbeha County, Mississippi, USA. Deer had year-round access to pelleted feed (20% crude protein; Purina AntlerMax Professional High Energy Breeder 59UB, Purina, St. Louis, Missouri, USA) *ad libitum* in 2 troughs located at opposite ends of each pen. Annual and perennial clovers (*Trifolium* spp.) and various native forbs and grasses grew as supplemental food throughout each pen. We obtained permission from private landowners to house additional male deer at satellite facilities near Macon, Noxubee County; Kosciusko, Attala County; Utica, Copiah County; and Morton, Scott County, Mississippi, USA. Satellite facilities consisted of 2 0.7-ha pens with similar feeding regimens as the MSU Deer unit.

We housed white-tailed deer of both sexes and varying ages in the 5 pens at the MSU Deer Unit. Adult males were present from October to April for breeding purposes. Mothers weaned fawns by mid-November and we removed fawns from the pens the following January each year. All individuals used in this study were born at the MSU Deer Unit.

### Data collection

We opportunistically assessed dominance hierarchies within each pen from December through April 2008–2011 using social interactions observed during daylight hours from 253 male and female white-tailed deer. Across 9 total pens we averaged 28 ± 8.4 deer per pen with a mean density of 34.7 ± 10.4 deer per hectare during November through April. We monitored interactions among females of breeding age (i.e., ≥ 1 year of age) continuously to ensure we documented maximum number of interactions [29]. Observation blocks ranged from 30 minutes to 4 hours. After exclusion of non-target animals (i.e., adult males and juvenile males and females ≤ 1 year of age) there were 11–26 females per pen (representing a total of 132 female years) that were available for dominance hierarchy analysis. After exclusion of non-target animals we averaged 15 ± 4.3 females per pen (Table 1) with a mean density of 17.9 ± 5.4 females per hectare across 9 pens. We observed females for 252 hours averaging 28 ± 8.2 hours per pen and recorded 2606 interactions. We placed about 900 g of triple-cleaned corn (Southern Seed & Feed, Macon, Mississippi, USA) in 3 piles about 1 meter apart at the beginning of each observation, creating a defendable resource to increase observable interactions [24, 30, 31]; however, we only observed about 1.4% of interactions at these piles.

**Table 1:** Year, pen, total interactions, total number of deer, directional consistency index, h (linearity), and h′ (linearity when unknown relationships are accounted for) associated with 9 dominance hierarchies of female white-tailed deer at the Mississippi State University captive research facility in Mississippi State, Mississippi, USA 2008–2011.

Year	Pen	Total Interactions Observed	Total Number of Deer	Directional Consistency Index	h	h'	*P*
2008–2009	1	78	14	0.87	0.27	0.37	0.057
	2	79	14	0.87	0.24	0.35	0.080
2009–2010	1^a^	567	13	0.78	0.41	0.44	0.018
	2	492	13	0.81	0.37	0.38	0.034
	3	193	11	0.69	0.50	0.55	0.009
	4	640	13	0.89	0.40	0.44	0.015
2010–2011	1^a^	154	13	0.84	0.38	0.44	0.033
	2	166	15	0.78	0.35	0.26	0.048
	4	237	26	0.90	0.18	0.25	0.003
	$\bar{X}$	290	15	0.83	0.34	0.39	N/A

^a^These pens contained the same deer during both sampling periods. The most dominant and most subordinate deer maintained the same rank in both years. However, 10 of 13 deer changed rank from 2009–2010 to 2010–2011.

We defined an interaction as an individual deer displaying dominant behaviors towards another deer. We defined interactions as independent when the females involved in a specific interaction assumed a non-dominant behavior (e.g., feeding, grooming) and were at least 1 body length apart from each other. Independent interactions may have included multiple behaviors [32]. We recorded 6 behaviors related to dominance: head high, ears back (stare from individual with ears flattened along neck), displacement, chase, foreleg kick (1 or more blows with forefoot), and rear and flail (rising on hind legs to kick with pedaling movement of forelegs; [33]) and recorded unique ear tag numbers of the aggressor and subordinate. We defined the loser as the deer that withdrew from the interaction.

We used Matman software (Noldus Information Technology version 1.1; Leesburg, Virginia, USA) to compare all pairwise animal interactions with at least 3 interactions between dyads to calculate a linear dominance hierarchy. We used Matman to test for linearity (h′ index) which accounts for individuals in a group with no direct interactions by comparing interactions of these individuals to other individuals with observed interactions [34]. This allowed us to include all females in our analysis. We used Matman to generate a randomization test to assess if the h′ index for each hierarchy was different from random. Linearity ranges from 0 (absence of linearity) to 1 (complete linearity; [34]); however, reported linearities involving ungulates range from 0.11 [14] to 0.94 [27]. We then used Matman to organize linear hierarchies by an iterative procedure (1000 randomizations) that ranks individuals by minimizing number of inconsistencies which occur when individual _j_ dominates individual _i_, when _j_’s rank was less than _i_’s [35]. Deer in our study established linear hierarchies within the 9 pens with an average h′ of 0.39 ± 0.09 and an average directional consistency index of 0.83 ± 0.07 (Table 1).

We chemically immobilized females for weighing (nearest 0.01 kg) prior to breeding during November 2008–2010. Mean maternal mass of females in our study was 48.8 ± 6.6 kg (n = 132). We used a 2:1 mixture of Telazol (Fort Dodge Animal Health, Fort Dodge, Iowa, USA) and xylazine HCl (Phoenix Scientific, St.Joseph, Missouri, USA) with an approximate dosage of 6.6 mg/kg via cartridge fired dart (Pneu-Dart Inc, Williamsport, Pennsylvania, USA). We reversed the effects of xylazine HCl with 0.125 mg/kg yohimbine HCl [36] or 4.0 mg/kg tolazoline HCl [37].

We systematically searched the entire deer pens for fawns daily starting on 1 June and we quit searching after the last pregnant mother gave birth each year. We uniquely marked fawns with medium plastic ear tags (Allflex, Dallas, Texas, USA), measured body mass (nearest 0.01 kg) using a digital vertical hanging scale (Pelouze, Bridgeview, Illinois, USA) and collected hair samples for parentage assignment. DNA Solutions (Oklahoma City, Oklahoma, USA) assigned parentage of fawns using DNA from hair based on a proprietary, non-statistical custom structured query language database known as the DNA Solutions Animal Solutions Manager (DASM). In the pairwise allele comparison DNA Solutions assigned parentage when they exclude all but 1 sire and 1 dam based upon a shared allele from each parent at all loci tested (B. G. Cassidy, DNA Solutions, personal communication). We determined litter size from parentage assignments. We found 119 litters resulting in 229 fawns with an overall mean of 1.8 ± 0.4 fawns/mother and mean birth mass of 2.6 ± 0.4 kg. Mothers produced 16 singletons, 96 litters of twins and 7 litters of triplets. Mean birth mass of singletons, twins, and triplets was 3.2 ± 0.4 kg, 2.6 ± 0.4 kg and 2.2 ± 0.7 kg, respectively. We caught all individuals used in this study as fawns.

### Data analyses

We used a mixed model analysis of variance in the lme4 package in Program R (R Development Core Team 2013 version 3.1.2) to quantify the relationship between fawn birth mass and adult body mass. We collected data for this analysis from male and female deer housed at the MSU Deer Unit and satellite facilities from 2005 to 2013. We categorized deer as mature at 3 years of age as more than 90% of maximum body mass is attained by then [38]. Body mass varied by source soil region [38] and potentially parentage (i.e., fawns born to wild caught mothers vs. fawns born from mothers born at the MSU Deer Unit; [39]), gender and litter size. Therefore, we accounted for this potential variation by including source soil region (a 3-level categorical variable) as a random effect. We also accounted for any potential cohort effects by including birth year as a random effect. We accounted for the potential influence of parentage, gender and litter size by including these variables as fixed effects.

**Fig 1:**
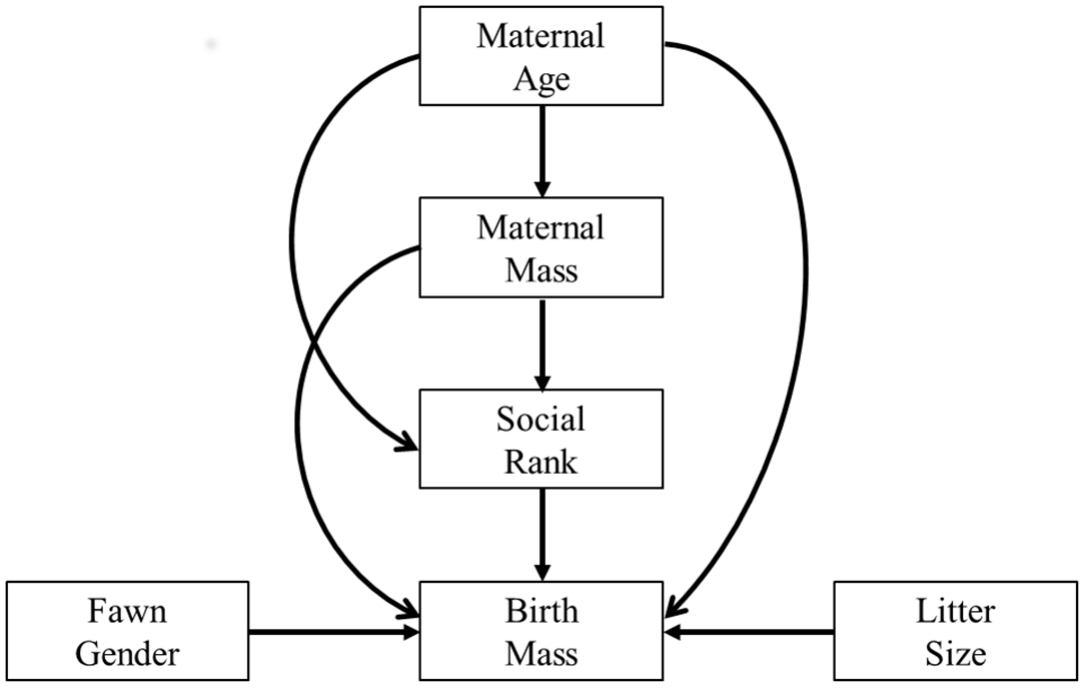
Conceptual model depicting all possible paths among maternal mass, maternal age and social rank for captive female white-tailed deer. Variables include maternal age, maternal body mass, social rank, litter size, fawn gender and fawn birth mass. Single headed arrows indicate a direct effect of one variable on another.

We assessed simple correlations among maternal body mass, maternal age, social rank and fawn birth mass using the cor.test function in Program R (R version 3.1.2) to justify using path analysis. Path analysis is a general form of multiple regression and is used to test theoretical relationships where multiple variables are correlated [40, 41]. However, due to the hierarchical structure of our data (repeated measurements of individuals across years) we tested our path models using Shipley’s d-sep (directional separation) procedure to test the hypotheses of conditional independence [42, 43]. For example, to test the conditional independence hypothesis of *X* and *Y* given variables *Z*_1_ and *Z*_2_, we obtained the null probability (p-value associated with the appropriate variable) that the slope of *X* was zero in a linear mixed model whose fixed structure is *Y*~*Z*_1_ + *Z*_2_ + *X* [44]. If our cause-effect paths among variables in our conceptual model (Fig 1) are correct, then the pattern of dependencies and (partial) independencies as shown by the path models are captured by the *k* mutually independent elements of Shipley’s [42, 43, 45] d-separation basis set of (partial) independencies and the statistic C = -2Ʃln(*p*_*i*_) using the *k* null probabilities (*p*_*i*_) associated with the basis set follows a X^2^ distribution with 2*k* degrees of freedom [44]. We calculated Akaike’s Information Criterion corrected for small sample size (AIC_c_) according to Shipley [46] and considered models within 2 ΔAIC_c_ competing [47]. We used the Bentler Comparative Fit Index (CFI) as an indication of model performance where values range from 0 (no fit) to 1 (perfect fit), with values ≥ 0.90 indicating good fit [41, 48, 49].

We included individual, year and source soil region as random effects when running mixed models for the d-sep analysis. Including these variables as random effects allowed us to account for repeated measurements of individuals, annual variations in both maternal and offspring body mass and regional variation in adult body mass. The effect of litter size on fawn birth mass is well established [4, 5]; therefore, we included litter size as a fixed effect. We also included fawn gender as a fixed effect to account for any variation in body mass between genders at birth. We fit mixed models with the restricted maximum likelihood using the lmer function in the lmer4 package in Program R (R Development Core Team 2013 version 3.1.2). We scaled social rank according to Côté [50] because number of deer varied across pens. We normalized adult and fawn body mass for use in all analyses by natural log transforming them. We determined that relationships among variables were linear.

We developed 14 models a priori based on white-tailed deer ecology. Our full model included all possible combinations of paths among maternal age, maternal body mass, social rank and fawn birth mass and served as our working hypothesis (Fig 1; [48]). We developed 13 additional models by removing individual paths among maternal age, maternal body mass, social rank and fawn birth mass while keeping the influence of fawn gender and litter size on fawn birth mass constant. The magnitude of a path coefficient (calculated as a standardized regression coefficient, *β*) indicates the degree of influence a variable has on another variable [48]. We used the same mixed model structure as described for the d-sep analysis to calculate path coefficients. We considered effects significant at α ≤ 0.05.

## Results

**Fig 2:**
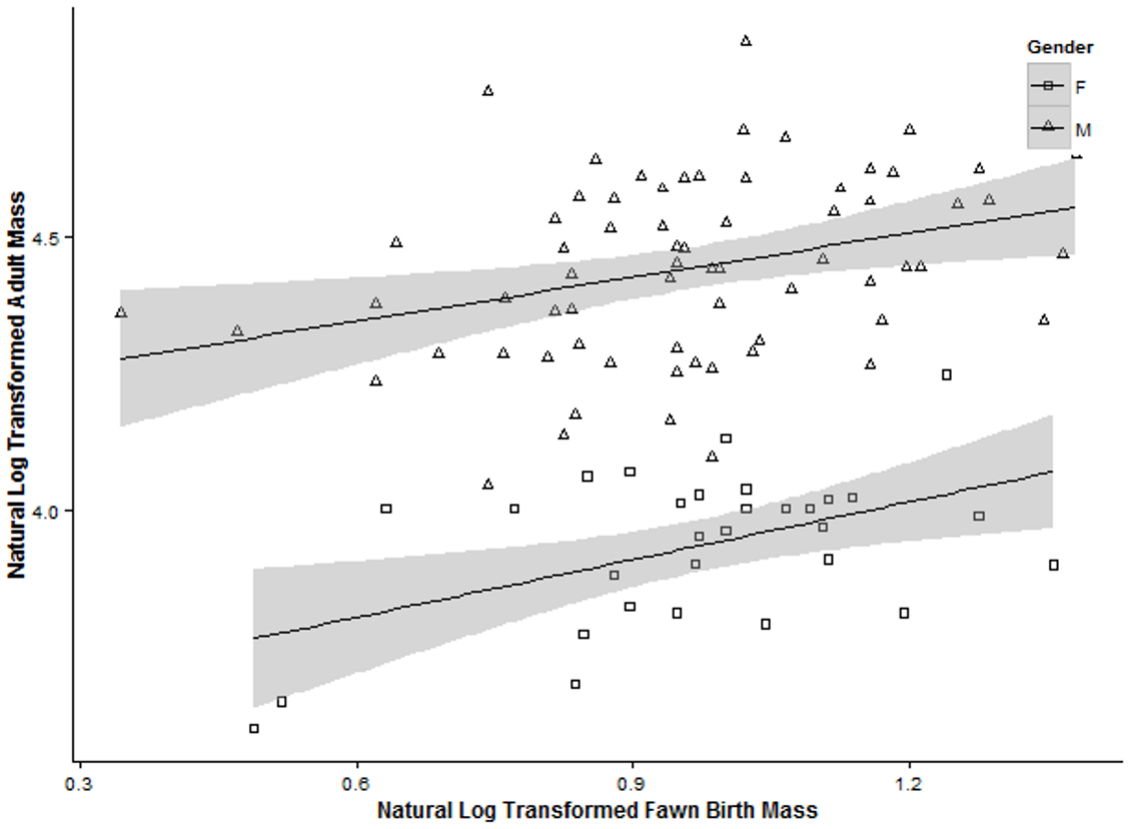
Scatterplot of the relationship between birth and adult body mass. Linear relationship of natural log transformed birth and adult body mass of male and female white-tailed deer at the Mississippi State University captive research facility, Mississippi State, Mississippi and satellite facilities located in Macon, Kosciusko, Utica and Morten, Mississippi, USA 2005–2013. Shaded area indicates the associated SEs.

Maternal mass, age, social rank and fawn birth mass were positively correlated, having *r* coefficients ranging from 0.171 to 0.321 (n = 229, Table 2). Fawn birth mass was a good predictor of adult body mass (*β* = 0.296, SE = 0.06, p < 0.001, n = 98; Fig 2).

**Table 2:** Correlation matrix summarizing the relationships among maternal age, maternal body mass, social rank and birth mass of female white-tailed deer at the Mississippi State University captive research facility in Mississippi State, Mississippi, USA 2008–2011. 95% CIs listed in parentheses below the Pearson correlation coefficient.

	Maternal Age	Maternal Mass	Social Rank	Birth Mass
Maternal Age				
Maternal Mass	*r* = 0.308 (0.16–0.421)			
Social Rank	*r* = 0.187 (0.059–0.309)	*r* = 0.321 (0.200–0.433)		
Birth Mass	*r* = 0.212 (0.085–0.33)	*r* = 0.318 (0.197–0.430)	*r* = 0.171 (0.043–0.294)	

**Fig 3:**
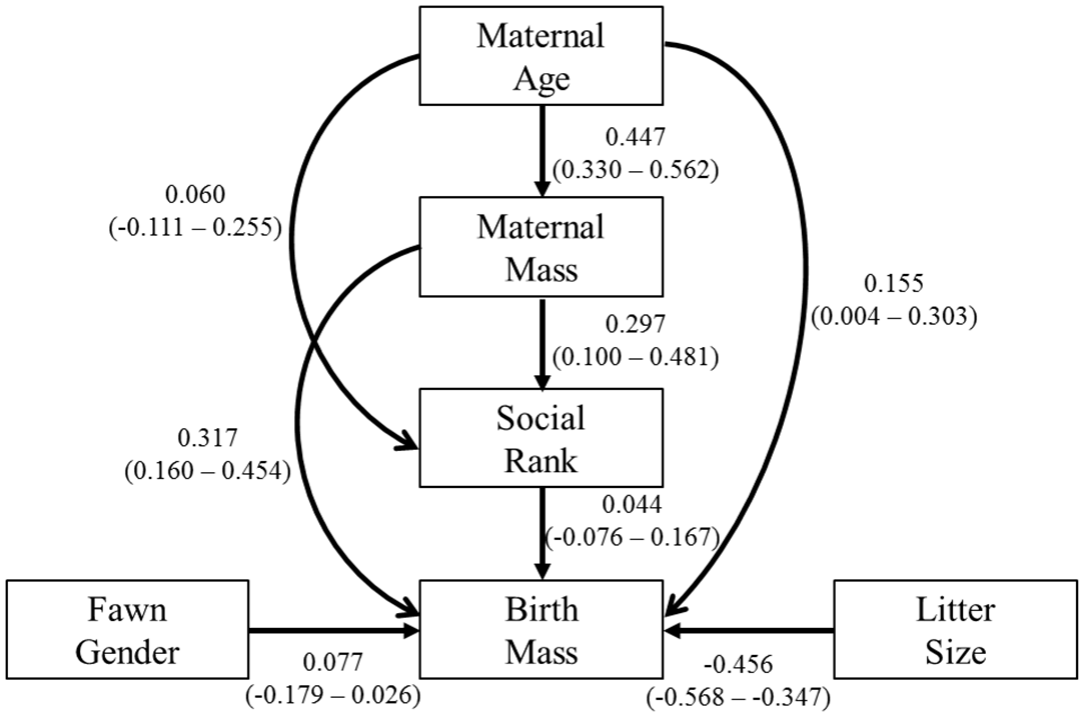
Conceptual diagrams of the top model for captive female white-tailed deer. Conceptual diagram of our AGE+MASS+RANK model depicting paths and effects of maternal age, maternal body mass, and social rank on birth mass. Single-headed arrows indicate direct paths between variables. Direct effects are standardized regression coefficients (*β*) and associated 95% CIs.

Five of the 14 models fit our data (C 17.89–27.40, DF 14–20, p ≥ 0.093) with a single model being superior (Table 3). Our best model shows direct paths from maternal age, maternal body mass, social rank, fawn gender and litter size to fawn birth mass (Fig 3). Litter size and maternal mass influenced fawn birth mass more than maternal age, fawn gender and social rank. Litter size displayed a moderate negative effect while maternal mass displayed a moderate positive effect (Fig 3). Maternal mass also influenced social rank more than maternal age (Fig 3). Although our top model depicts a direct path from fawn gender and social rank to fawn birth mass, as well as a direct path from maternal age to social rank, these relationships must be interpreted with caution as their 95% confidence intervals overlap 0 (Fig 3). We tested 7 claims of independence in our best model with t values ranging from 0.293 to 0.767 and null probabilities ranging from 0.004 to 0.977 (Table 4).

**Table 3:** Model selection results for fitted models describing the influence of maternal age, maternal body mass and social rank on fawn birth mass using AIC_c_ for white-tailed deer at the Mississippi State University captive research facility at Mississippi State, Mississippi, USA 2008–2011. *K* indicates the number of parameters and *w*_*i*_ indicates the AIC_c_ weight. Bentler Comparative Fit Index (CFI) indicates the performance of proposed models. The model name indicates which variables had direct paths to fawn birth mass. Our top model is represented in Fig 3. Diagrams of the other models can be found in S1 Appendix.

Model #	Model	K	ΔAICc	*w*i	CFI
12	AGE + MASS + RANK	14	0.00	0.53	0.88
4	MASS + RANK	12	2.69	0.14	0.83
8	MASS	11	2.76	0.13	0.82
6	MASS^a^	12	3.24	0.11	0.83
2	MASS + RANK^a^	13	3.65	0.09	0.84

^a^Indicates that models differed only in how maternal age affected social rank

**Table 4:** Tests of conditional independence of the basis sets implied by the path model in Fig 3. Variables: 1 (age), 2 (maternal body mass), 3 (social rank), 4 (fawn birth mass), 5 (litter size), 6 (fawn gender).

Independence Claim	*t* value	Null Probability (*p*_*i*_)
1_||_5|{}	0.767	0.449
1_||_6|{}	0.757	0.453
2_||_5|{1}	-0.216	0.829
2_||_6|{1}	-1.124	0.263
3_||5_|{1,2}	-0.028	0.977
3_||_6|{1,2}	0.243	0.808
5_||_6|{}	-2.934	0.004

## Discussion

Previous research has shown that offspring mass can directly [17, 23] and indirectly (reviewed in [51]) increase inclusive fitness over a range of environmental conditions. However, for this advantage to hold one must assume the positive correlation between birth and adult mass persists regardless of environmental conditions (reviewed in [51]). Our results support this previous research and our hypothesis that birth mass would positively influence adult body mass. Therefore, production of heavy offspring is beneficial to a mother by potentially improving her fitness.

The positive direct effect of maternal body mass on fawn birth mass supports our hypothesis. This could be explained by larger mothers having proportionally more reserves to mobilize during gestation than smaller mothers and thus are better able to produce heavy offspring [52]. However, in our study the need to mobilize reserves during gestation was minimized by year-round access to high-quality food *ad libitum*. Another possibility is larger mothers acquire more available food than smaller mothers, as displayed in cotton rats (*Sigmodon hispidus*; [52]). These authors reported heavier animals had a greater food intake rate and a greater ability to use food resources compared to lighter animals. Heavy mothers may be able to increase their food intake by defending the best foraging areas where food is distributed heterogeneously from lighter mothers.

Dominance may be the mechanism by which heavy mothers improve their production of heavy offspring by facilitating increased access to food when limited [24]. Our data show direct paths from maternal age and body mass to social rank. Increased social rank improves access to limited food which in turn positively influences maternal body condition and reproductive success [24–26]. The overall importance of social rank position would be expected to diminish in the presence of abundant food resources as indicated by the weak total effect of social rank on fawn birth mass in our model. This weak effect of social rank on fawn birth mass supports our hypothesis as well as previous studies such as Taillon and Côté [27] and Vogel [28] where social rank did not influence potential benefits associated with increased food acquisition when food was abundant. Nevertheless, our results suggest that social rank may be a mechanism used by heavy mothers to gain access to resources.

The ability to monopolize food using dominance behaviors may be important for certain reproductive strategies. For example, ungulates are generally considered capital breeders but species such as white-tailed deer may differ where they occur on the capital/income breeding continuum [53]. Obligatory fat storage occurs during winter for white-tailed deer in temperate regions which improves survival [54]. This fixed physiological response supports gestation and is consistent with a capital breeding strategy. However, fetal development is minimal in the first 2 trimesters in which stored reserves would be used [55]. Fetal development rapidly increases in the third trimester which also coincides with changes in plant phenology (i.e., “spring green-up”) when food resources are reappearing on the landscape [55, 56]. The relationship between fetal development and spring green-up suggests that white-tailed deer also display tendencies of an income breeder, similar to roe deer which are also classified as income breeders [57]. Thus, dominance may serve as a mechanism that allows heavy females to obtain greater relative access to limited food during early spring and produce heavier offspring compared to light females. However, this relationship likely varies as winter severity and winter food availability differs across the range of white-tailed deer.

Age is associated with 2 factors related to the production and rearing of heavy offspring. First, age represents reproductive classification (i.e., immature or mature). White-tailed deer increase body mass to 3-years of age but can conceive as early as 6 months of age depending on food quality and abundance [38, 58]. Deer less than 3-years old would not be expected to produce as heavy of offspring compared to older individuals as they still bear the energetic costs of growth and therefore must partition food resources towards growth and reproduction [4, 5, 7]. Our results show that once the relationship between age and maternal body mass is accounted for that maternal body mass was clearly more important than age in the production of heavy offspring over the range of ages in our population. Secondly, age can be considered a proxy for experience [59]. As such, variation in experience associated with the 4-year age range in our study appears to minimally influence production of heavy offspring. However, the relationship between maternal age and offspring birth mass may strengthen when food is limited and/or distributed heterogeneously on the landscape as age, and thus experience, may impart advantages during food acquisition. Maternal experience is also important in the rearing of offspring. Ozoga and Verme [59] reported fawns from “older” white-tailed deer mothers had greater survival than fawns of “younger” mothers, though their results may have been confounded as they did not indicate whether they accounted for the strong relationship between maternal age and maternal mass. Nevertheless, experience likely plays a more important role in the rearing of heavy offspring rather than in the production of heavy offspring.

We report moderately strong relationships between maternal body mass and fawn birth mass indicating that other variables likely influence the production of heavy offspring. For example, prior-year reproductive success and maternal care may also affect offspring birth mass by influencing early life characteristics such as growth rate and weaning mass [60, 61]. Future research should include multiple maternal (body mass, body condition, age, prior-year reproductive success and maternal care) and early life characteristics (parturition date and litter size) in a comprehensive framework to partition how they individually and collectively influence birth mass and whether these relationships persist to adulthood and subsequently affect their offspring (inter-generational fitness).

## Conclusion

Maternal body mass of female white-tailed deer had more influence on fawn birth mass than maternal age and social rank. Although these maternal characteristics are inter-related, maternal phenotype appears to affect production of heavy offspring more than maternal experience and behavior. However, dominance may facilitate the positive relationship between maternal body mass and fawn birth mass as maternal body mass positively influenced social rank position. This in turn could impart an inter-generational relationship as heavier mothers gain greater access to limited food resources and thus are able to produce heavy offspring which grow into heavy adults with greater reproductive success than light offspring. The relationship between fawn birth mass and future reproductive success has not yet been determined for white-tailed deer. Knowing what mechanisms enable mothers to produce heavy and potentially more reproductively successful offspring would allow ecologists to better understand how successful mothers increase their inclusive fitness.

## Supporting Information

S1 Appendix Diagrams of alternate models listed in Table 3. (ZIP)

S1 Dataset Maternal body mass, maternal age, maternal social rank, litter size and average birth mass of offspring. (XLSX)

S2 Dataset Individual body masses throughout each animals life. (XLSX)
