# Supplementary figures and images for: Contrasting the Effects of Maternal and Behavioral Characteristics on Fawn Birth Mass in White-Tailed Deer
Source: PLoS One. 2015 Aug 19;10(8):e0136034. doi: 10.1371/journal.pone.0136034 (PMC4546060; doi:10.1371/journal.pone.0136034)

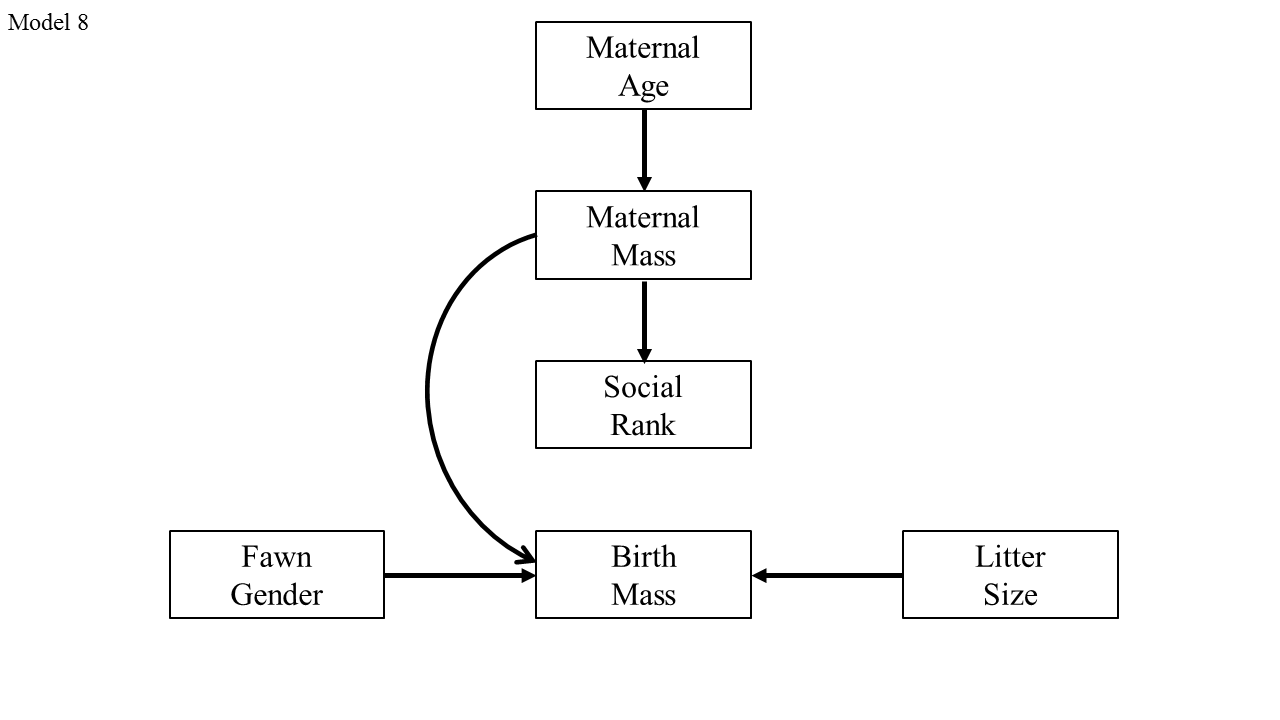

Supplement: S1 Appendix — (ZIP) [file pone.0136034.s001.zip › Model 8.TIF]

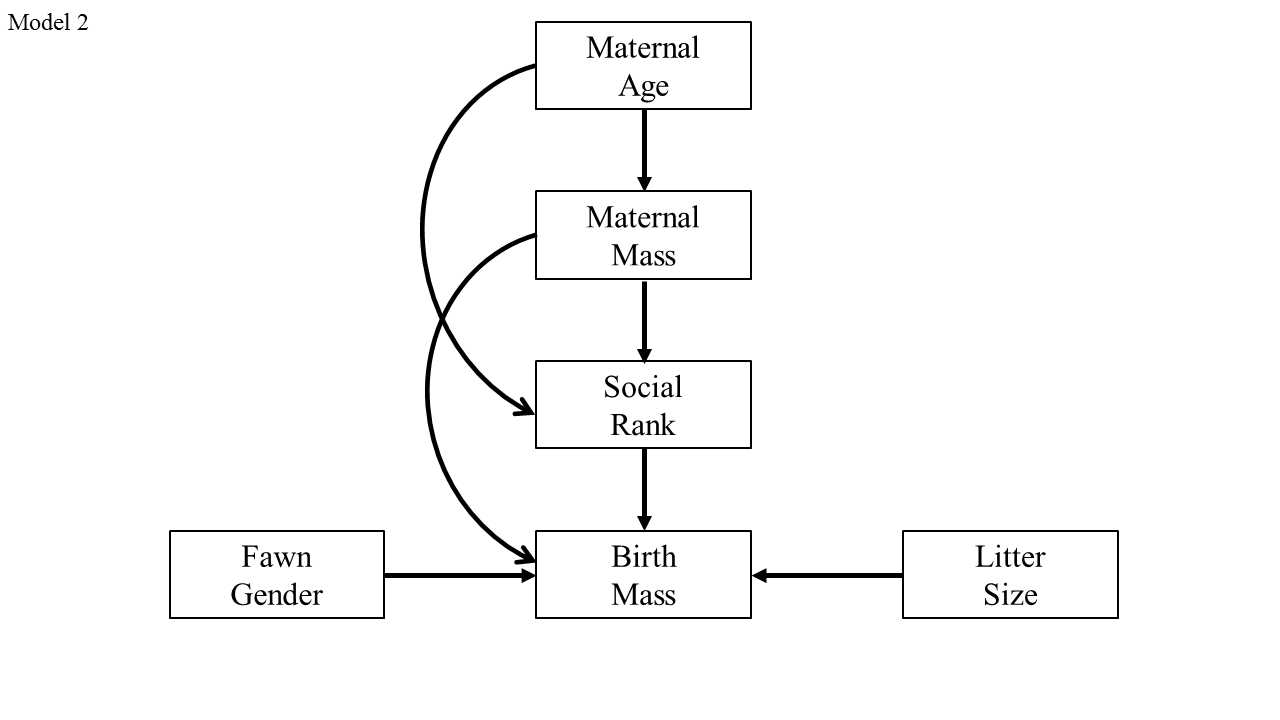

Supplement: S1 Appendix — (ZIP) [file pone.0136034.s001.zip › Model 2.TIF]

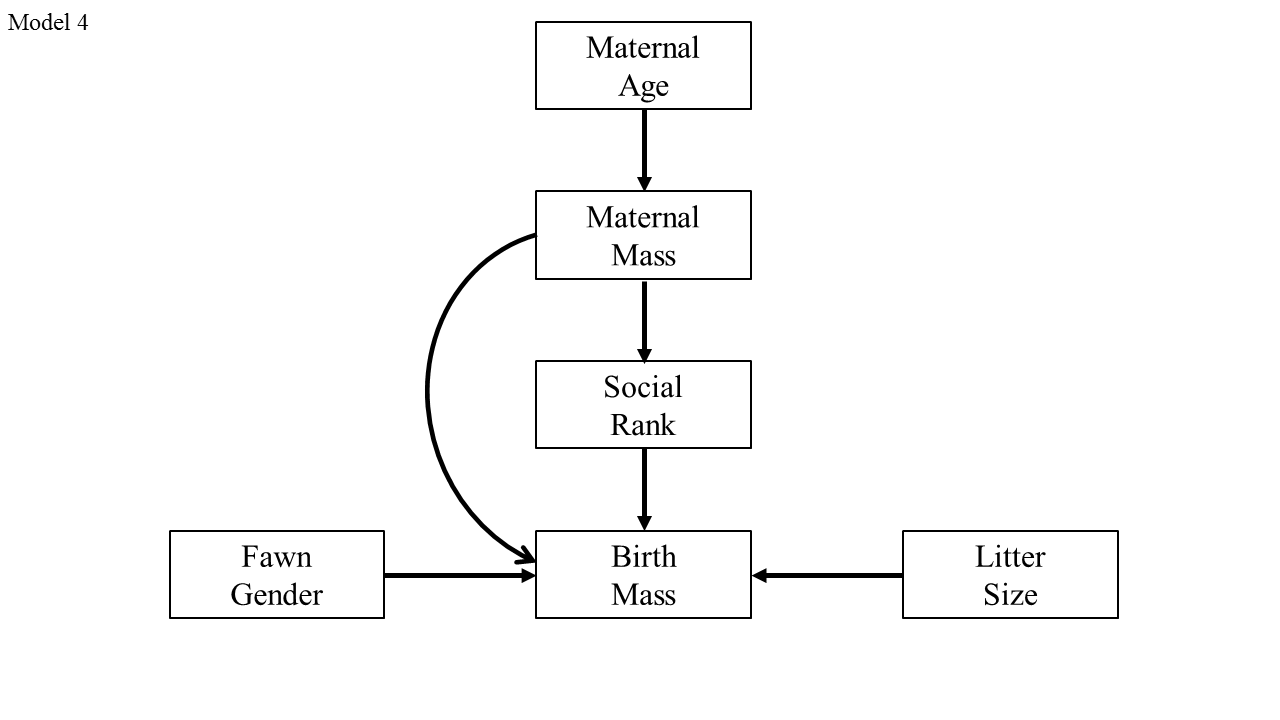

Supplement: S1 Appendix — (ZIP) [file pone.0136034.s001.zip › Model 4.TIF]

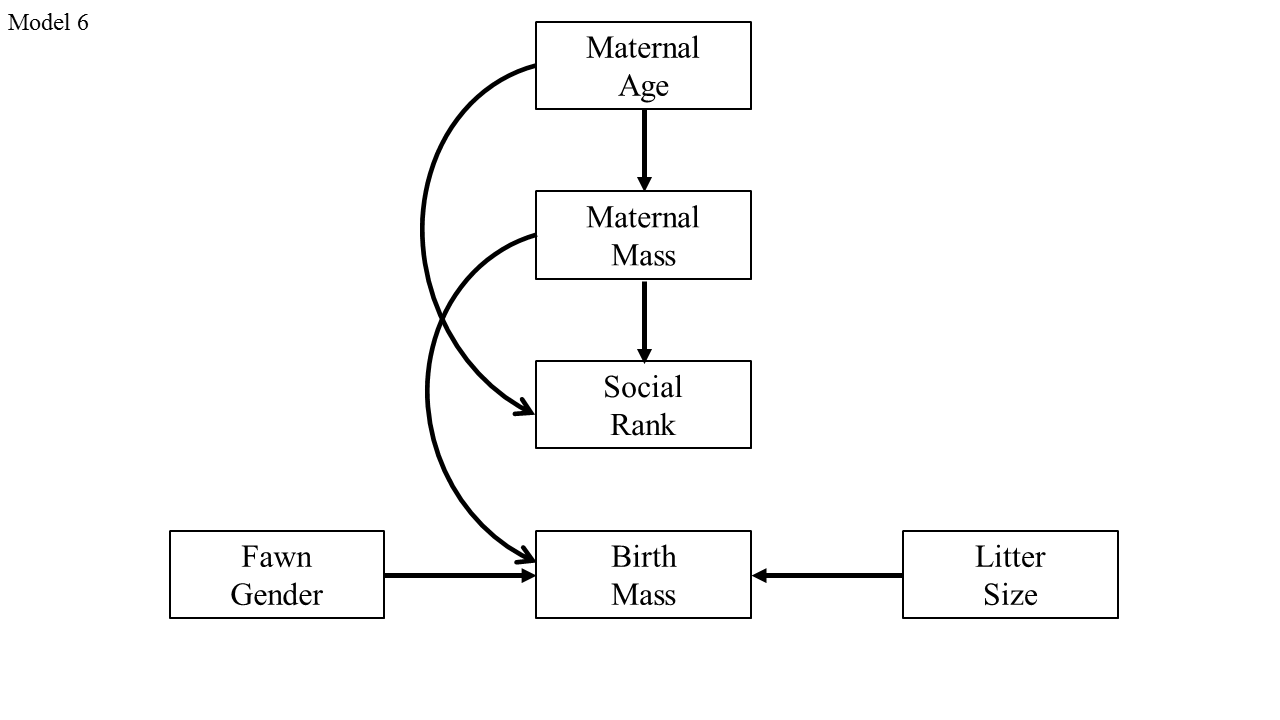

Supplement: S1 Appendix — (ZIP) [file pone.0136034.s001.zip › Model 6.TIF]
